# Evaluating behavioral responses of nesting lesser snow geese to unmanned aircraft surveys

**DOI:** 10.1002/ece3.3731

**Published:** 2017-12-25

**Authors:** Andrew Barnas, Robert Newman, Christopher J. Felege, Michael P. Corcoran, Samuel D. Hervey, Tanner J. Stechmann, Robert F. Rockwell, Susan N. Ellis‐Felege

**Affiliations:** ^1^ Department of Biology University of North Dakota Grand Forks ND USA; ^2^ Division of Vertebrate Zoology American Museum of Natural History New York NY USA

**Keywords:** *Anser caerulescens*, behavior, disturbance, drone, nest camera, noninvasive, unmanned aircraft system, waterfowl

## Abstract

Unmanned aircraft systems (UAS) are relatively new technologies gaining popularity among wildlife biologists. As with any new tool in wildlife science, operating protocols must be developed through rigorous protocol testing. Few studies have been conducted that quantify the impacts UAS may have on unhabituated individuals in the wild using standard aerial survey protocols. We evaluated impacts of unmanned surveys by measuring UAS‐induced behavioral responses during the nesting phase of lesser snow geese (*Anser caerulescens caerulescens*) in Wapusk National Park, Manitoba, Canada. We conducted surveys with a fixed‐wing Trimble UX5 and monitored behavioral changes via discreet surveillance cameras at 25 nests. Days with UAS surveys resulted in decreased resting and increased nest maintenance, low scanning, high scanning, head‐cocking and off‐nest behaviors when compared to days without UAS surveys. In the group of birds flown over, head‐cocking for overhead vigilance was rarely seen prior to launch or after landing (mean estimates 0.03% and 0.02%, respectively) but increased to 0.56% of the time when the aircraft was flying overhead suggesting that birds were able to detect the aircraft during flight. Neither UAS survey altitude nor launch distance alone in this study was strong predictors of nesting behaviors, although our flight altitudes (≥75 m above ground level) were much higher than previously published behavioral studies. *Synthesis and applications*: The diversity of UAS models makes generalizations on behavioral impacts difficult, and we caution that researchers should design UAS studies with knowledge that some minimal disturbance is likely to occur. We recommend flight designs take potential behavioral impacts into account by increasing survey altitude where data quality requirements permit. Such flight designs should consider *a priori* knowledge of focal species’ behavioral characteristics. Research is needed to determine whether any such disturbance is a result of visual or auditory stimuli.

## 1 INTRODUCTION

Unmanned aircraft systems (UAS) have gained popularity as a tool for research in wildlife ecology, particularly in ornithological studies (Anderson & Gaston, 2013; Christie, Gilbert, Brown, Hatfield, & Hanson, 2016). These technologies are relatively novel, yet have evolved rapidly to fit a wide variety of avian research questions and applications. Early work focused on the feasibility of using UAS for bird‐related research and addressed questions of detectability (Jones, 2003; Jones, Pearlstine, & Percival, 2006; Watts et al., 2008, 2010). Colony and flock size estimates have been conducted for staging flocks of geese (Chabot & Bird, 2012), breeding populations of black‐headed gull *Chroicocephalus ridibundus* (Sardà‐Palomera et al., 2012), penguins (*Pygoscelis sp*.) in Antarctica (Goebel et al., 2015; Ratcliffe et al., 2015), and sandhill crane *Grus canadensis* flocks along their migratory routes (USGS 2011). UAS have been used for monitoring coastal habitat use of mixed waterbird flocks (Drever et al., 2015), measuring habitat quality for threatened least bitterns *Ixobrychus exilis* (Chabot & Bird, 2013; Chabot, Carignan, & Bird, 2014), and understanding habitat selection of lesser kestrels *Falco naumanni* (Rodríguez et al., 2012). Other applications used UAS to conduct maritime surveillance in a marine‐protected area used by seabird colonies (Brooke et al., 2015) and to evaluate powerline electrocution risks for nesting raptors (Mulero‐Pázmány, Negro, & Ferrer, 2013).

Another popular application of UAS is the ability to monitor birds during their reproductive period at multiple spatial scales. Unmanned aircraft have been deployed at the landscape level to survey greater sage‐grouse *Centrocercus urophasianus* leks (Hanson, Holmquist‐Johnson, & Cowardin, 2014) and estimate nesting density of common terns *Sterna hirundo* (Chabot, Craik, & Bird, 2015). Other studies have shown UAS to be an effective method for determining nesting status of several raptor species including osprey *Pandion haliaetus*, bald eagle *Haliaeetus leucocephalus*, ferruginous hawk *Buteo regalis*, red‐tailed hawk *Buteo jamaicensis* (Junda, Greene, & Bird, 2015), and Stellar's sea eagle *Haliaeetus pelagicus* (Potapov, Utekhina, McGrady, & Rimlinger, 2013). Weissensteiner, Poelstra, and Wolf (2015) found that UAS can be efficiently used to save time in checking nest contents of canopy‐nesting birds by eliminating the need for surveyors to climb trees for such inspections. Other authors have noted similar benefits of using UAS for studying birds, such as the relatively low cost, ease of use, and time savings (Anderson & Gaston, 2013; Jones et al., 2006; Watts et al., 2010).

Across the variety of applications, the most commonly cited benefit of UAS for wildlife research is that these technologies have low impact or have a reduced disturbance effect when compared to manned aircraft surveys or researchers on the ground (Christie et al., 2016; Ward, Stehn, Erickson, & Derksen, 1999). The low impact factor of UAS on birds is poorly documented and is often based on anecdotal observations or general impressions of behavior (Brooke et al., 2015; Chabot & Bird, 2012; Goebel et al., 2015; Grenzdörffer, 2013; Kudo, Koshino, Eto, Ichimura, & Kaeriyama, 2012; Potapov et al., 2013; Ratcliffe et al., 2015; Weissensteiner et al., 2015). Some studies have attempted to document behavioral responses using dedicated spotters (Chabot et al., 2015; Drever et al., 2015; Hanson et al., 2014) or post hoc analysis of imagery (Dulava, Bean, & Richmond, 2015; Sardà‐Palomera et al., 2012), although they are not inclusive of a study design that rigorously evaluates behavioral responses. Several studies have attempted to quantify bird behavior in response to UAS but often lack controls for baseline behavioral patterns or use flight designs that do not represent standard survey protocols such as line transects (Junda, Greene, Zazelenchuk, & Bird, 2016; McEvoy, Hall, & McDonald, 2016; Rümmler, Mustafa, Maercker, Peter, & Esefeld, 2015; Vas, Lescroël, Duriez, Boguszewski, & Grémillet, 2015; Weimerskirch, Prudor, & Schull, 2017). More importantly, these designs do not account for habituation of individuals to repeated flights, thus masking any behavioral signals that may be apparent to novel stimuli but are lost with repeated exposures. The increasing trend of using UAS for avian research warrants a robust quantification of potential impacts to the wildlife species being studied, which is currently lacking in the field of UAS for wildlife studies (Christie et al., 2016; Crutsinger, Short, & Sollenberger, 2016; Hodgson & Koh, 2016; Smith et al., 2016).

Several recent reviews of UAS used for wildlife research have concluded that UAS surveys result in minimal disturbance, although this is likely dependent on a variety of factors such as aircraft type, flight patterns, and taxa (Borrelle & Fletcher, 2017; Chabot & Bird, 2015; Christie et al., 2016). Mulero‐Pázmány et al. (2017) found that birds were more prone to behavioral responses [during UAS surveys] than other taxa and expressed the need for standardized experiments to evaluate causes of disturbance during UAS surveys. Quantification of behavioral impacts can be difficult given that observers on the ground are likely to miss short‐lived or ephemeral behaviors. Collected videos of individual birds allow for the review and characterization of a wider spectrum of behaviors than is available to real‐time observers. The objective of this study is to remotely characterize and quantify the behavioral responses of nesting waterfowl to unmanned aircraft surveys using nest‐camera footage. Specifically, we examine (1) if behaviors are affected by the presence of UAS survey flights and (2) which factors associated with UAS flights may play a role in bird behavior.

## 2 METHODS

### 2.1 Study species and area

Given the increased use of UAS for monitoring colonial nesting birds, flights and behavioral observations were conducted on lesser snow geese *Anser caerulescens caerulescens* (hereafter LSGO) during incubation. The widespread distribution of LSGO nesting colonies in remote arctic locations makes this species a good candidate for future UAS studies and applications.

Study sites were located in Wapusk National Park (WNP) in northeastern Manitoba, and research was based out of a remote field camp (N 58.725388°, W −93.464288°). Topography in this region is uniformly low‐lying with little overhead cover for nesting waterfowl. With the exception of researcher activity, there is restricted access to the vast majority of WNP, leaving these study sites relatively free of anthropogenic influences during the waterfowl incubation season.

### 2.2 Behavior monitoring

Ground searches were conducted to locate nests approximately halfway through the incubation period to avoid disrupting birds during nest‐initiation. Initiation was determined by floating goose eggs in water and measuring the position eggs held when submerged (Westerskov, 1950). Nests were randomly selected for behavioral monitoring provided individual nests were greater than 75 m away from the nearest monitored nest as measured by handheld Garmin eTrex‐20 and 64S GPS (Garmin, Olathe, KS). We established a minimum nest‐distance to increase the likelihood that individual nest behaviors were independent of neighboring nest behaviors. For ease of flight operations, nests were grouped into clusters with a 500‐m buffer between groups to ensure UAS flights over groups did not inadvertently affect birds not intended to be flown over.

To monitor the behavior of nesting birds during UAS surveys, video surveillance cameras were deployed at nests to record continuous video (Burr, Robinson, Larsen, Newman, & Ellis‐Felege, 2017). Cameras were powered by 12‐V, 36‐amp batteries and equipped with 32‐GB SD cards, allowing individual systems to operate and record for 5–9 days without need of researcher maintenance and minimizing disturbance to birds. Cameras were set up 1 m from the nest, and a 25‐m cable connected them to a DVR housed inside a camouflaged, waterproof box and connected to the battery. The bulk of camera equipment (DVR, batteries, etc.) was stored far from the nest to reduce potential influences on the hen's behavior and also reduce the chance of attracting curious predators.

Data collection and monitoring of waterfowl nests were authorized by Canadian Wildlife Service Research and Collection Permit 16‐MB‐SC001 and 11‐MB‐SC001, Wapusk National Park WAP‐2015‐18670 and WAP‐2016‐21419 and the University of North Dakota Institutional Animal Care and Use Committee approvals #A3917‐01, Protocols 1505‐2 and 1505‐10.

### 2.3 Flight operations

Flights were conducted using a Trimble UX5 (color: black, wingspan: 100 cm, weight: 2.5 kg, cruise speed: 80 km/hr, see Figure S1), a fixed‐wing rear‐propelled aircraft powered by removable lithium polymer batteries (14.8 V, 6000 mAh). UX5 takeoffs are initiated using an elastic catapult launcher. Once the flight area has been covered, the UX5 begins its descent and eventually belly lands as the aircraft lacks skid gear of any kind. Takeoffs and landings were carried out at a minimum of 325 m from monitored nests. All flight plans were preprogrammed line transects using Trimble Access Aerial Imaging V2.0.00.40 (Trimble, Sunnyvale, CA) and georeferenced in real time using the UX5′s built‐in GPS system with 80% overlap of adjacent images. Flight path directory and angle of approaches are dictated by environmental factors such as wind speed and direction. Still images are automatically taken with a Sony NEX‐5R 16.1‐MP camera (Sony Corporation of America, New York, NY) along flight paths. Imagery is downloaded following completion of a flight and used to create landscape mosaics from which habitat characteristics and nest density can be evaluated.

Between June 11–18, 2015 and June 3–16, 2016, flights were conducted at altitudes of 75, 100 and 120 m above ground level (AGL). Flight paths were designed to fly over groups of monitored nests at specified altitudes, such that other monitored nests (nontargets) were not flown over at the same time. A control group of monitored nests was never flown over with the UAS to serve as baseline behavioral comparisons.

Unmanned aircraft systems flight operations for this research were approved by Transport Canada in accordance with a Special Flight Operations Certificate (File: 5802‐11‐302, ATS: 14‐15‐00067822 and 15‐16‐00058646, RDIMS: 10610691 and 11717338) and by Wapusk National Park with WAP‐2015‐18846. Further, the UND Unmanned Aircraft System Research Compliance Committee reviewed human privacy and data management protocols for the project (Approved April 10, 2015).

### 2.4 Video review and behavioral classifications

**Figure 1:**
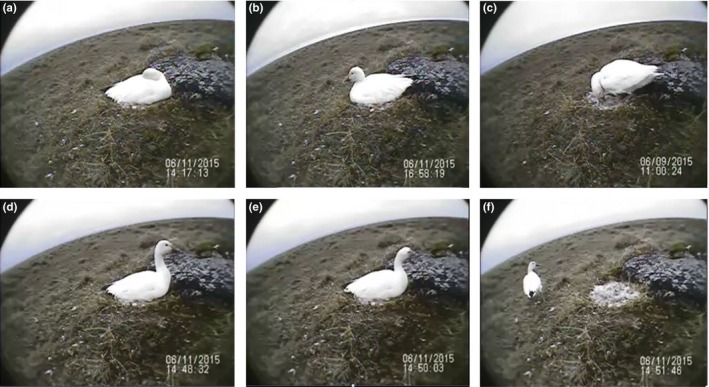
Behavioral classifications for nesting waterfowl (LSGO pictured above). (a) Resting, (b) Low Scan, (c) Nest Maintenance, (d) High Scan, (e) Head Cock, (f) Off Nest

SD cards were retrieved from monitored nests during nest checks and after completion of UAS flights. Video files were downloaded to a hard drive. A single observer (AB) reviewed video using Windows Media Player (Microsoft, Seattle, WA). Time stamps on video files were matched with flight operation times, and behavioral observations were made continuously from 30 min prior to takeoff and until 60 min after the aircraft had landed. We selected 30 min prior to takeoff to include more than the team's average setup time of 20 min. We selected 1 hr after landing to allow time to examine bird behavior to residual effects of the flight. Behaviors were classified into six broad categories: resting, nest maintenance, low scanning, high scanning, head‐cocking, and off nest (Figure 1). Resting was comprised of mostly sleeping but also included heads tucked back into the body while still awake. Nest maintenance involved activities such as contributing vegetation to nest bowls, egg‐turning, or self‐preening. Low scanning was a very low activity behavior wherein birds seemed to be passively observing their environment, in stark contrast to high scanning in which birds were attentively observing by means of rapid head movement or raised necks. Head‐cocking was distinctly different from high scanning and was classified by birds tilting their head to observe overhead (Video S1). Off nest was recorded upon birds standing and leaving their nest. We further categorized off nest to include whether or not birds covered their eggs with insulating down before leaving the nest. As individual flight times varied throughout flight operations, behaviors were calculated as relative proportions rather than absolute time durations.

### 2.5 Data analysis

To determine the effects of flight operations on nesting birds, we constructed generalized linear mixed models examining the proportion of time birds engaged in each of the six different behavior classifications using PROC GLIMMIX in SAS Studio 3.7 (Cary, NC). Each behavior was analyzed as a separate response to test for effects of factors on specific components of behavior. To facilitate the use of linear models, we logit transformed (log(y/[1‐y])) our proportion data which is bounded between 0 and 1 (Warton & Hui, 2011). To ensure logit‐transformed data did not contain any undefined values, we used an empirical logit transformation by adding or subtracting a small value (0.0001) to proportion values of 0 or 1, respectively (Iles, Salguero‐Gómez, Adler, & Koons, 2016; Peterson, Rockwell, Witte, & Koons, 2013). To avoid model dredging and allow comparison of a restricted number of models, we selected factors of interest and relevant possible interactions prior to statistical analyses (Burnham & Anderson, 2002; Zuur, Ieno, & Elphick, 2010).

As we were first interested in whether UAS flights played any role in bird behaviors, we constructed candidate models [Equation (1)] with the fixed effects *day* of flight operation (categorical with two levels: flight or no‐flight), treatment *group* (categorical with two levels: surveyed birds and control birds with no flights overhead), and the interaction term *day* × *group*. To incorporate dependency among observations in the same nest and period of observation, we used *nest_id* and *flight_id* as random effects with an autoregressive covariance structure to account for decay in correlation with increased distance and time between observations (Barnett, Koper, Dobson, Schmiegelow, & Manseau, 2010).

$${\text{Response}}_{\mathrm{ijk}}∼\text{Gaussian}\left(\mu_{\mathrm{ijk}}\right)$$

$$E\left({\text{Response}}_{\mathrm{ijk}}\right)=\mu_{\mathrm{ijk}}$$

$$\text{Logit}\left(\mu_{\mathrm{ijk}}\right)={\text{Day}}_{\mathrm{ijk}}+{\text{Group}}_{\mathrm{ijk}}+{\text{Day}}_{\mathrm{ijk}}\times {\text{Group}}_{\mathrm{ijk}}+{\text{Nest}}_{i}+{\text{Flight}}_{j}$$

$${\text{Nest}}_{i}∼\text{Gaussian}\left(0,\sigma^{2}\right)$$

(1) $${\text{Flight}}_{j}∼\text{Gaussian}\left(0,\sigma^{2}\right)$$

A separate set of models was then constructed to examine which factors within UAS flight operations influence bird behavior on flight days only [Equation (2)]. Fixed effects were treatment *group* (categorical with two levels: surveyed birds and control birds with no flights overhead), flight *altitude* (categorical with four levels: 75, 100, 120 m above group, and a 0 category for control birds), and *launch distance* (Euclidean distance of individual nest to UAS launch site, range 325–2,100 m). Also included was the categorical fixed effect of *period* within flight operation with three levels: 30 min before UAS launch (Pre), the duration of the flight (Air), and 60 min postlanding (Post). We included the interaction terms *group* × *period* as we felt it was import to examine how behaviors between groups vary depending on whether the aircraft was in the air or not. For both model sets, we were unable to include predator presence as a covariate due to our long distances from focal nests. As with our previous models, *nest_id* and *flight_id* were included as random effects with an autoregressive covariance structure.

$${\text{Response}}_{\mathrm{ijk}}∼\text{Gaussian}\left(\mu_{\mathrm{ijk}}\right)$$

$$E\left({\text{Response}}_{\mathrm{ijk}}\right)=\mu_{\mathrm{ijk}}$$

$$\begin{array}{cc} \text{Logit}(\mu_{\mathrm{ijk}}) & ={\text{Group}}_{\mathrm{ijk}}+{\text{Altitude}}_{\mathrm{ijk}}+\text{Launch}\;{\text{Distance}}_{\mathrm{ijk}}+{\text{Group}}_{\mathrm{ijk}}\times {\text{Period}}_{\mathrm{ijk}}\\ & +{\text{Nest}}_{i}+{\text{Flight}}_{j} \end{array}$$

$${\text{Nest}}_{i}∼\text{Gaussian}\left(0,\sigma^{2}\right)$$

(2) $${\text{Flight}}_{j}∼\text{Gaussian}\left(0,\sigma^{2}\right)$$

In all models, Response_*ijk*_ is the *k*th observation at Nest_*i*_ (*i *=* *1…25) and Flight_*j*_ (*j *=* *1…13). Individuals in treatment *group* were only included in the control group if they had never been flown over with the UAS. For all model sets, we included a null model that included the intercept and random effects only. Models were evaluated using Akaike Information Criterion (AICc) for small sample sizes (Akaike, 1973; Burnham & Anderson, 2002). Model assumptions were assessed by visually examining probability plots of the residuals for global models of each response behavior (Burnam et al., 2012; Suraci, Clinchy, Dill, Roberts, & Zanette, 2016). Because linear models are relatively robust to nonnormality, visual inspections are a good method to assess whether a candidate set of models adequately describes the variability of data (Raudenbush & Bryk, 2002; Zuur et al., 2010). We assessed model fit by examining the deviance of candidate models in comparison with null deviance. For top models, we back‐transformed estimates and 95% confidence limits to the original data scale for presentation (Jørgensen & Pedersen, 1998; Vander Yacht et al., 2016).

## 3 RESULTS

We conducted 26 LSGO flights in 2015 and 2016 and deployed cameras for behavioral observations at 32 LSGO nests. Not all flights and nests were included in the analyses due to logistic or technical difficulties (e.g., nest predation, nest‐camera failures). Of the birds flown over, we collected behavioral data for 18 LSGO from 13 flights. Control data were collected from 7 LSGO nests. Average UAS flight duration was 32 min (range: 13–42 min).

### 3.1 Effect of UAS flight presence

**Figure 2:**
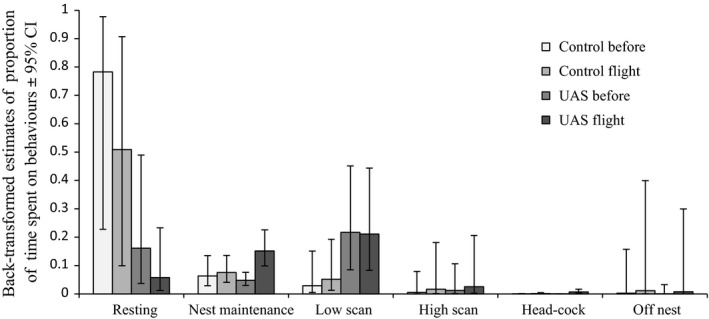
Back‐transformed estimates and 95% confidence intervals of proportion of time LSGO spent on individual behaviors within treatment groups (Control vs. UAS) and between days (Before vs. Flight). Behavioral data from 67 observations at 25 nests across 13 UAS flights

Our best model (lowest AICc score) for all behaviors was the interactive model of *day* × *group* (Table 1). For all behavioral responses, the top model possessed >65% AICc weight, and the second best model had a minimum ∆AICc >2.0 (see Appendix S1). Visual inspection of the global model residuals did not reveal substantial deviations from normality, which is expected as a result of the logit‐transformed data (Appendix S1). We back‐transformed estimates of behavior proportions and 95% confidence limits (Figure 2) for each behavior. In control birds, sleeping decreased on days of UAS flight operations while all other behaviors increased. For birds in the UAS treatment group, sleeping and low scanning decreased on flight days, while nest maintenance, high scanning, head‐cocking, and off‐nest behaviors increased (Figure 2). In all cases of LSGO leaving the nest during observation periods, birds covered their nest with insulating down.

**Table 1:** Beta estimates from top model (*day* × *group*) for the proportion of time^a^ spent on behaviors of nesting LSGO relative to whether or not a UAS survey flight occurred (*day* where UAS = birds flown over, CTRL = birds not flown over) and treatment (*group*). Estimates obtained from 67 observations at 25 nests across 13 UAS flights

Behavior	*w*	Intercept β ± *SE*	UAS × Day before^b^ β ± *SE*	UAS × Flight day^b^ β ± *SE*	CTRL × Flight day^b^ β ± *SE*
Resting	0.721	1.2817 ± 1.2308	−2.9303 ± 1.4037	−4.0790 ± 1.4037	−1.2454 ± 0.9626
Nest Maintenance	0.798	−2.6915 ± 0.4102	−0.2941 ± 0.4762	0.9673 ± 0.4762	0.1821 ± 0.5213
Low Scan	0.651	−3.5310 ± 0.8857	2.2476 ± 1.0566	2.2148 ± 1.0566	0.6231 ± 0.9838
High Scan	0.683	−5.2956 ± 1.3980	0.8755 ± 1.2612	1.6563 ± 1.2612	1.1973 ± 1.1458
Head‐Cock	0.854	−8.5943 ± 0.7616	0.1109 ± 0.8842	3.5994 ± 0.8842	1.9785 ± 0.9680
Off Nest	0.786	−5.9746 ± 2.1128	−1.4177 ± 1.4067	1.1342 ± 1.4067	1.5029 ± 1.4014

a Note β and *SE* estimates remain on logit‐transformed scale.

b Baseline comparisons are to the control group of birds the day before flight operation.

### 3.2 Effect of factors within UAS flight operations

The top two models for all behaviors were either the model of *altitude* + *period* or the interactive model *group* × *period*. Nest maintenance, high scanning, and head‐cocking had high support for their top model *group* × *period*, with weights of 0.85, 0.75, and 0.92, respectively (see Appendix S2). Resting, low scanning, and off nest had low ∆AICc and similar weights between the two top models, indicating that similar amounts of variation are explained by both models (Burnham & Anderson, 2002). Because the covariate *altitude* had a built‐in group component (0‐m altitude for birds not flown over [controls]), this suggests that treatment group plays some role in both top models, as does *period*. For simplicity, we report results for *group* × *period* as the best model for explaining behavioral responses on flight days (Table 2).

**Table 2:** Estimates from the model (*group* × *period*) for the proportion of time^a^ spent on behaviors of nesting LSGO during UAS survey flight days relative to treatment *group* where (UAS = birds flown over, CTRL = birds not flown over) and flight operation *period* where (PRE = 30 min before launch, AIR = the period in which the UAS was airborne, and POST = 1 hr after landing). Estimates obtained from 114 observations at 25 nests across 13 UAS flights

Behavior	*w*	Intercept β ± *SE*	CTRL × AIR^b^β ± *SE*	CTRL × POST^b^β ± *SE*	UAS × PRE^b^β ± *SE*	UAS × AIR^b^β ± *SE*	UAS × POST^b^β ± *SE*
Resting	0.721	−0.6063 ± 1.9195	−0.8059 ± 1.6957	−0.8428 ± 1.6957	−1.6995 ± 2.0630	−4.0738 ± 2.0630	−3.1931 ± 2.0630
Nest Maintenance	0.798	−4.3628 ± 0.9116	−1.1186 ± 1.2352	1.5177 ± 1.2352	0.1261 ± 1.0981	1.2784 ± 1.0981	2.6975 ± 1.0981
Low Scan	0.651	−4.9940 ± 1.2461	−1.0968 ± 1.0964	2.2643 ± 1.0964	1.5884 ± 1.3376	2.2023 ± 1.3376	4.0999 ± 1.3376
High Scan	0.683	−5.9157 ± 1.2153	−0.6418 ± 0.9291	1.5720 ± 0.9291	0.1849 ± 1.2308	1.4040 ± 1.2308	1.4409 ± 1.2308
Head‐Cock	0.854	−8.9180 ± 0.7296	2.1538 ± 1.0318	1.3113 ± 1.0318	0.8319 ± 0.8995	3.7308 ± 0.8995	0.5481 ± 0.8995
Off Nest	0.786	−6.3329 ± 1.5767	−0.4442 ± 1.4328	0.9327 ± 1.4328	−2.0708 ± 1.6124	−0.8054 ± 1.6124	0.7456 ± 1.6124

a Note β and *SE* estimates remain on logit‐transformed scale.

b Baseline comparisons are to the control group of birds during the period before the aircraft is in the air (CTRL × PRE).

Resting and nest maintenance behaviors decreased in both groups once the aircraft was in the air (Table 3). In the control groups low and high scanning decreased during the Air period, but increased during the Post period. In the UAS group, scanning behaviors increased throughout flight operations. For both the control and the UAS group, head‐cocking increased while the aircraft was in the air, although this increase was greater in the UAS group. Mean estimates for head‐cocking in control birds increased from 0.0001 to 0.0012 when the aircraft launched, while birds flown over increased from 0.0003 to 0.0056, suggesting that birds were engaging in increased overhead vigilance regardless if the UAS was directly overhead. The amount of time birds spent off nest increased in the postflight period for both groups, again this increase was greater in the UAS group. Large confidence intervals around estimates suggest high variability in individual behavioral response.

**Table 3:** Back‐transformed estimates and 95% confidence intervals from the model (*group* × *period*) for the proportion of time spent on behaviors of nesting LSGO during UAS survey flight days relative to treatment *group*, and flight operation *period*. Estimates obtained from 114 observations at 25 nests across 13 UAS flights

Behavior	CTRL × PRE	CTRL × AIR	CTRL × POST	UAS × PRE	UAS × AIR	UAS × POST
Resting						
μ	0.3529	0.1959	0.1901	0.0906	0.0092	0.0219
95% CI	0.0118 < μ < 0.9614	0.0053 < μ < 0.9176	0.0051 < μ < 0.9148	0.0059 < μ < 0.6245	0.0006 < μ < 0.1341	0.0013 < μ < 0.2719
Nest Maintenance						
μ	0.0126	0.0041	0.0549	0.0142	0.0438	0.1591
95% CI	0.0021 < μ < 0.0726	0.0007 < μ < 0.0249	0.0094 < μ < 0.2631	0.0038 < μ < 0.0517	0.0120 < μ < 0.1473	0.0477 < μ < 0.4166
Low Scan						
μ	0.0067	0.0023	0.0612	0.0321	0.0578	0.2903
95% CI	0.0006 < μ < 0.0750	0.0002 < μ < 0.0264	0.0054 < μ < 0.4382	0.0053 < μ < 0.1712	0.0098 < μ < 0.2762	0.0616 < μ < 0.7179
High Scan						
μ	0.0027	0.0014	0.0128	0.0032	0.0109	0.0113
95% CI	0.0002 < μ < 0.0294	0.0001 < μ < 0.0157	0.0012 < μ < 0.1274	0.0005 < μ < 0.0202	0.0017 < μ < 0.0652	0.0018 < μ < 0.0675
Head‐Cock						
μ	0.0001	0.0012	0.0005	0.0003	0.0056	0.0002
95% CI	0.0000 < μ < 0.0006	0.0003 < μ < 0.0049	0.0001 < μ < 0.0021	0.0001 < μ < 0.0009	0.0020 < μ < 0.0157	0.0001 < μ < 0.0007
Off Nest						
μ	0.0018	0.0011	0.0045	0.0002	0.0008	0.0037
95% CI	0.0001 < μ < 0.0394	0.0000 < μ < 0.0256	0.0002 < μ < 0.0944	0.0000 < μ < 0.0026	0.0001 < μ < 0.0093	0.0003 < μ < 0.0422

Distance between the nest and launch site ranged from 325 to 2,100 m, and we suspected launch distances would influence behaviors. However, we did not find this to be an important predictor of behaviors as *launch distance* was the least supported model for all behaviors (Appendix S2). Model deviances are reported in Appendices S1 and S2.

## 4 DISCUSSION

Our study addressed key weaknesses of previous work by quantifying behavioral observations of a waterfowl species using replication and controls. Here, we also included baseline observations to demonstrate changes in behavior, a metric lacking in previous studies (Rümmler et al., 2015; Vas et al., 2015). Our results demonstrate there is a quantifiable change in behavior of nesting waterfowl during UAS surveys compared to nonsurvey days. However, we acknowledge there was considerable variation in responses between individual birds, and as such results should be interpreted with caution. On days with surveys, birds in both groups spent less time resting at the nest and were more likely to participate in active behaviors suggesting higher levels of alertness. Previous studies have shown anthropogenic disturbances reduce time spent feeding by geese, resulting in a net loss of energetic intake (Bélanger & Bédard, 1990; Owens, 1977). Several species of geese have been shown to lose 11.4–27.1% of their body mass by the end of incubation. Additional energetic losses through reduced feeding or increased activity at the nest have the potential to reduce reproductive fitness and should be avoided if possible. Our results suggest that the increased activity during UAS surveys could result in changes in energetics and should be a focus of future research and consideration.

Arctic nesting geese heavily invest in nest attendance by spending greater than 90% of their time on the nest during incubation (Reed, Hughes, & Gauthier, 1995; Thompson & Raveling, 1987). Here, we documented slightly increased time spent off nest on days with UAS surveys, which puts LSGO nests at risk of predation by arctic foxes *Vulpes lagopus* and aerial predators (Samelius & Alisauskas, 2001). Although again, off‐nest responses were highly variable. We did not observe any predation events during any observation periods, and in all off‐nest events, birds covered their eggs with insulating down before leaving the nest. Although aerial predators are frequently spotted in our field site, we were unable to account for predator presence near nests in this study due to the limited field of view for nest cameras to focus on individual behavior. It is possible that increased disturbance by UAS has the indirect effect of increasing an individual's vigilance, reducing the ability of predators to ambush nesting hens, although future analyses would be required to determine the long‐term effects of UAS surveys on nest success. In contrast, investigator disturbance by researchers on the ground significantly increases the risk of nest attack in a greater snow goose colony (Bêty & Gauthier, 2001). When birds flushed off nest because of researchers, only 32–47% of birds covered their eggs with nest material, leaving the nest exposed (Bêty & Gauthier, 2001).

During UAS flights, the period of flight operations when the aircraft was flying accompanied increased levels of head‐cocking, indicating birds were noticing the aircraft as it surveyed. Similar aerial vigilance behaviors have been noted in Antarctic birds surveyed with a microcopter UAS (Rümmler et al., 2015; Weimerskirch et al., 2017) and several species of waterfowl surveyed with various UAS models (McEvoy et al., 2016). However, the increased aerial vigilance was observed in both the UAS and control treatment groups suggesting that either (1) birds were visually aware of the aircraft at >500 m lateral distance or (2) birds were responding to an auditory disturbance produced by the aircraft. While the indication that birds are aware of the aircraft, the biological relevancy of this behavior is likely minimal because the highest estimate of head‐cocking accounted for less than 2% of the observation period. The small proportion of time is likely due to the ephemeral nature of head‐cocking events (Video S1), although we feel it is a strong indication of birds being able to detect the unmanned aircraft. Discerning between visual and auditory disturbance of UAS surveys is difficult and future work should address this; however, we suspect the geese are detecting the sound of the aircraft and then searching for the source of the sound.

Differences in size and wing profiles of different fixed‐wing UASs can influence the behavioral responses of waterfowl (McEvoy et al., 2016; Mulero‐Pázmány et al., 2017). Our small unmanned fixed‐winged aircraft may resemble the silhouette of raptor species, leading to a higher perceived threat to bird species that are typical prey of raptors, thus leading to potential disturbance issues (McEvoy et al., 2016). Future experimentation with shapes resembling common raptors and nonpredatory birds should be planned to further inform the design of low‐disturbance aircraft. Using rotary wing, UAS may decrease the likelihood of these predator resemblance responses, although such aircraft are accompanied with higher dB output and shorter battery endurance for flight operations (McEvoy et al., 2016). Increased dB levels have been shown to elicit increased disturbance and alert behaviors in sea birds (Brown, 1990) and nesting osprey (Trimper et al., 1998), although small UAS operations conducted higher than 100 m AGL have reduced impacts from noise disturbance (Mulero‐Pázmány et al., 2017). For our future purposes of estimating nesting LSGO densities, rotary‐wing quadcopters are likely unable to cover the large areas given the limited endurance of these platforms.

Launch distance (and thus direct influence of human operators) was not in our top models influencing behavior as expected, although most launch distances were substantially farther than previous behavioral studies which were often within 100 m from the study organisms (Junda et al., 2016; Rümmler et al., 2015; Vas et al., 2015). Several observations of LSGO near the launch site (<50 m) indicated that individuals were more alert to our presence upon arrival though quickly habituated. Thus, our launch distance limited inference on human activity near the nests, but may be an important consideration in future UAS protocols aimed at being less invasive.

Our study found survey altitude alone to be a poor predictor of behavioral changes, contrary to previous studies which generally found increased responses with lower survey altitude (Rümmler et al., 2015; Vas et al., 2015). However, our lowest flight altitude was greater than the highest altitude presented in these previous studies, likely rendering differences in our survey altitudes to be negligible for nesting birds. There likely exists a threshold altitude where wildlife responds proportionately to any decreases in UAS survey altitude, although we did not find such any such threshold. Thus, we suggest using UAS sensors that enable users to fly at least 75 m AGL to further reduce the risk of impacting species of interest while maintaining sufficient data quality. Understanding data resolution needs and selecting an appropriate sensor to meet those needs at specific altitudes during planning will be an important survey design consideration to minimize wildlife disturbances.

Although it is clear that UAS surveys result in some minimal changes in waterfowl behavior, this should not dissuade the use of these novel technologies for ecological applications surrounding waterfowl and other wildlife. Results from this study demonstrate that UAS offer a relatively low‐impact survey method for surveying nesting waterfowl. The diversity of UAS models currently available makes generalizations on behavioral impacts difficult. We caution that researchers should design UAS studies with the knowledge that some disturbance is likely to occur and make efforts to minimize it. Further, it should be noted that different aircraft models and flight designs will be needed to fit species‐specific data needs and that some aircraft may be inappropriately utilized if prior considerations for study design are not taken. It is up to individual researchers to balance the need for high‐quality data with the potential for species impact. As such, *a priori* knowledge of a focal species should be taken into consideration before selection for a UAS study to avoid potential negative impacts.

Future research is needed to determine whether any such disturbance is a result of visual or auditory stimuli, and how development of UAS for wildlife research should proceed. Direct comparisons of disturbance between UAS and ground‐based surveys are needed, but any future studies should be designed to match actual survey protocols that would be used for data collection, rather than methods that would not be reproduced as a part of standard UAS use. However, as UAS technology and practices are still developing, potential users should cautiously consider the appropriate aircraft and flight design to meet data needs before adopting these tools.

## DATA ACCESSIBILITY

Data available upon request and will be submitted to Dryad at publication.

## CONFLICT OF INTEREST

None declared.

### AUTHOR CONTRIBUTIONS

Experimental design was conceived by A. Barnas, R. Newman, M. Corcoran., R. Rockwell., and S. Ellis‐Felege; data analysis and writing were conducted by A. Barnas and S. Ellis‐Felege; all authors contributed with field logistics, data acquisition, and editing of this manuscript. All authors gave final approval for publication.
