# Supplementary material for: Evaluating behavioral responses of nesting lesser snow geese to unmanned aircraft surveys
Source: Ecol Evol. 2017 Dec 25;8(2):1328–38. doi: 10.1002/ece3.3731 (PMC5773326; doi:10.1002/ece3.3731)
Supplement: Supplementary file 1 [file ECE3-8-1328-s001.pdf]

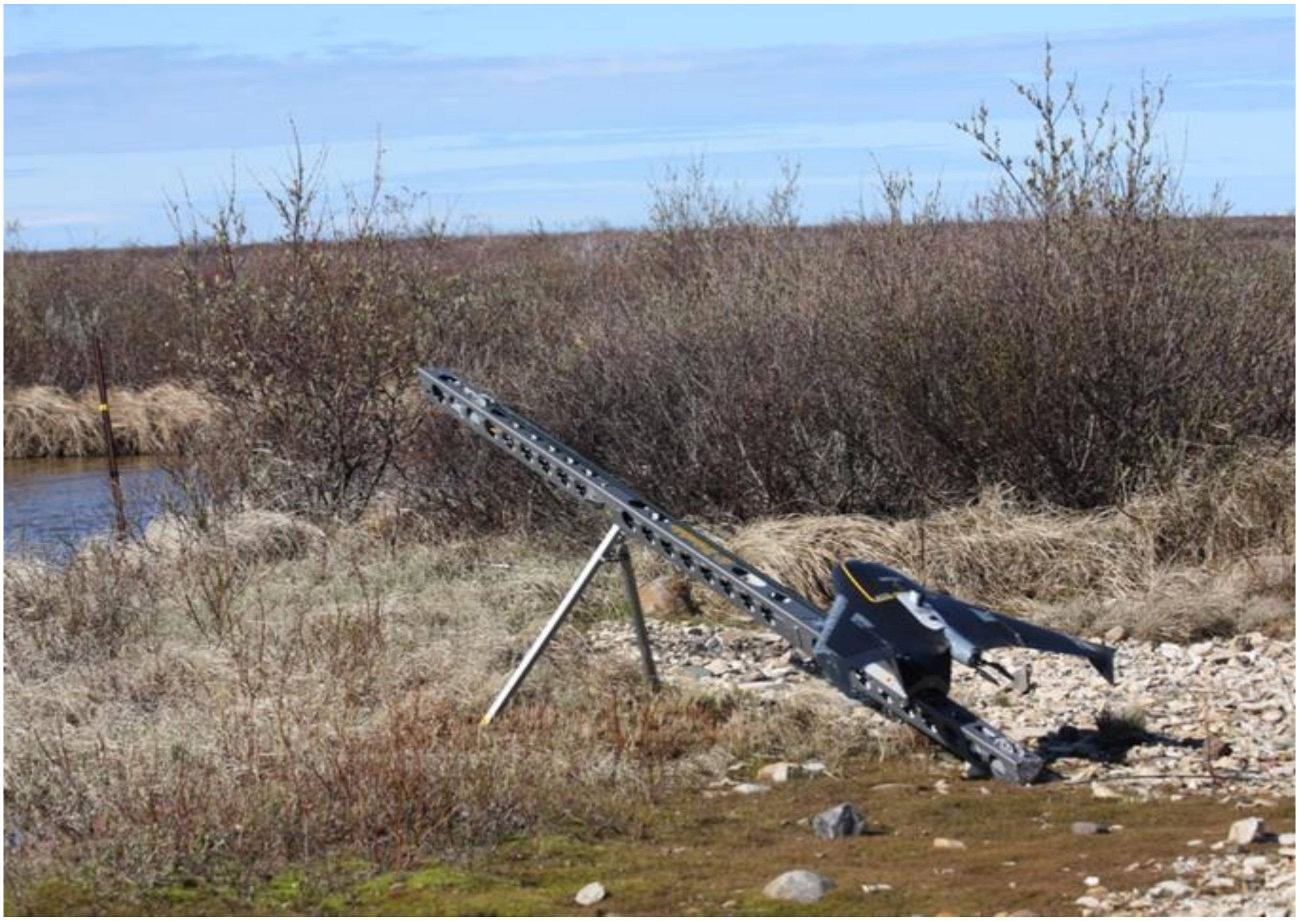

Supplementary Figure 1 The Trimble UX5 (http://uas.trimble.com/ux5) on its elastic catapult launcher. Photo credit: Susan Ellis-Felege.
