# Supplementary material for: Evaluating behavioral responses of nesting lesser snow geese to unmanned aircraft surveys
Source: Ecol Evol. 2017 Dec 25;8(2):1328–38. doi: 10.1002/ece3.3731 (PMC5773326; doi:10.1002/ece3.3731)
Supplement: Supplementary file 2 [file ECE3-8-1328-s002.pdf]

Appendix S1 Candidate models with their corresponding AICc scores, weights (w), and deviances for each behaviour response (Resting, Nest Maintenance, Low Scan, High Scan, Head Cock and Off Nest) of LSGO on days before and during UAS surveys. "*day*"= flight vs no flight, "*group*"= flown over vs control birds, "null" = intercept and random effects only.

Model	AICc	ΔAICc	w	Deviance
Resting				
Day*Group	328.71	0	0.721	322.31
Day Group	330.78	2.07	0.256	324.38
Group	336.5	7.79	0.015	330.11
Day	337.93	9.22	0.007	331.54
Null	342.89	14.18	0.001	336.5
Nest Maintenance				
Day*Group	212.62	0	0.798	208.42
Day Group	216.48	3.86	0.116	212.28
Day	217.14	4.52	0.083	212.94
Group	225.61	12.99	0.001	221.42
Null	225.7	13.08	0.001	221.51
Low Scan				
Day*Group	306.57	0	0.651	300.16
Day Group	309.02	2.45	0.191	302.62
Group	309.65	3.08	0.139	303.26
Day	314.76	8.19	0.011	308.36
Null	315.37	8.8	0.008	308.98
High Scan				
Day*Group	328.2	0	0.683	321.79

Day Group	330.72	2.52	0.194	324.32
Day	332.89	4.69	0.065	326.5
Group	333.74	5.54	0.043	327.34
Null	335.75	7.55	0.016	329.36
Head Cock				
Day*Group	290.59	0	0.854	286.39
Day Group	294.42	3.83	0.126	290.22
Day	298.1	7.51	0.020	293.9
Group	321.54	30.95	0.000	317.34
Null	323.47	32.88	0.000	319.28
Off Nest				
Day*Group	347.61	0	0.786	341.2
Day Group	350.85	3.24	0.156	344.45
Day	352.9	5.29	0.056	346.51
Group	359.88	12.27	0.002	353.48
Null	362.67	15.06	0	356.28
