# Supplementary material for: Evaluating behavioral responses of nesting lesser snow geese to unmanned aircraft surveys
Source: Ecol Evol. 2017 Dec 25;8(2):1328–38. doi: 10.1002/ece3.3731 (PMC5773326; doi:10.1002/ece3.3731)
Supplement: Supplementary file 3 [file ECE3-8-1328-s003.pdf]

Appendix S2 Candidate models with their corresponding AICc scores, weights (w), and deviances for each behaviour response type (Resting, Nest Maintenance, Low Scan, High Scan, Head Cock and Off Nest) of LSGO during UAS surveys. "*group*"= flown over vs control birds, "*altitude*"= altitude of survey 75m, 100m, 120m or control (no flight overhead), "*launch distance*"= distance from aircraft launch to nest observed, "*period*"= period of flight (PRE, AIR, POST), "*global*" = *group* + *altitude* + *launch distance* + *period*, "*null*"= intercept and random effects only.

Model	AICc	ΔAICc	w	Deviance
Resting				
Altitude + Period	682.45	0	0.537	676.22
Group x Period	682.85	0.4	0.440	676.61
Alt	689.43	6.98	0.016	683.2
Global	692	9.55	0.005	685.76
Period	694.72	12.27	0.001	688.5
Group	696.7	14.25	0	690.48
Null	701.66	19.21	0	695.44
Launch distance + Period	703.26	20.81	0	697.04
Launch distance	710.21	27.76	0	703.99
Nest Maintenance				
Group x Period	580.31	0	0.852	574.08
Altitude + Period	583.94	3.63	0.139	577.71
Period	590.02	9.71	0.007	585.91
Global	592.85	12.54	0.002	588.74
Alt	594.05	13.74	0.001	589.94
Group	597.91	17.6	0	593.8
Launch distance + Period	598.26	17.95	0	594.15

Null	602.14	21.83	0	598.03
Launch distance	610.6	30.29	0	606.49
Low Scan				
Group x Period	588.93	0	0.500	582.7
Altitude + Period	588.94	0.01	0.497	582.71
Global	599.89	10.96	0.002	593.66
Period	601.51	12.58	0.001	595.29
Alt	608.23	19.3	0	602.21
Launch distance + Period	612.57	23.64	0	606.34
Group	614.36	25.43	0	608.14
Null	619.74	30.81	0	613.52
Launch distance	630.9	41.97	0	624.68
High Scan				
Group x Period	560.57	0	0.753	554.34
Altitude + Period	562.91	2.34	0.234	556.68
Alt	569.93	9.36	0.007	563.71
Period	571.01	10.44	0.004	564.78
Global	573.15	12.58	0.001	566.92
Group	575.75	15.18	0	569.53
Null	578.08	17.51	0	571.86
Launch distance + Period	582.34	21.77	0	576.12
Launch distance	589.42	28.85	0	583.19
Head Cock				
Group x Period	536.88	0	0.924	532.77
Altitude + Period	542.12	5.24	0.067	535.89

Period	546.39	9.51	0.008	542.28
Global	553.3	16.42	0	547.06
Launch distance + Period	558.15	21.27	0	554.04
Alt	564.29	27.41	0	560.18
Group	567.38	30.5	0	563.27
Null	569.02	32.14	0	564.91
Launch distance	580.78	43.9	0	576.67
Off Nest				
Altitude + Period	636.96	0	0.453	630.73
Group x Period	637.06	0.1	0.431	630.83
Global	639.83	2.87	0.108	633.6
Alt	646.57	9.61	0.004	640.34
Period	646.83	9.87	0.003	640.6
Launch distance + Period	653.03	16.07	0	646.8
Group	653.69	16.73	0	647.47
Null	656.32	19.36	0	650.1
Launch distance	662.48	25.52	0	656.25
